# A need for One Health approach – lessons learned from outbreaks of Rift Valley fever in Saudi Arabia and Sudan

**DOI:** 10.3402/iee.v4.20710

**Published:** 2014-02-04

**Authors:** Osama Ahmed Hassan, Clas Ahlm, Magnus Evander

**Affiliations:** 1 Department of Epidemiology and Diseases Control, Public Health Institute, Federal Ministry of Health, Khartoum, Sudan; 2 Department of Clinical Microbiology, Infectious Diseases, Umeå University, Umeå, Sweden; 3 Department of Clinical Microbiology, Virology, Umeå University, Umeå, Sweden

**Keywords:** Rift Valley fever, Saudi Arabia, Sudan, One Health, outbreak, climate, mosquito, trade ban

## Abstract

**Introduction:**

Rift Valley fever (RVF) is an emerging viral zoonosis that impacts human and animal health. It is transmitted from animals to humans directly through exposure to blood, body fluids, or tissues of infected animals or via mosquito bites. The disease is endemic to Africa but has recently spread to Saudi Arabia and Yemen. Our aim was to compare two major outbreaks of RVF in Saudi Arabia (2000) and Sudan (2007) from a One Health perspective.

**Methods:**

Using the terms ‘Saudi Arabia’, ‘Sudan’, and ‘RVF’, articles were identified by searching PubMed, Google Scholar, and web pages of international organizations as well as local sources in Saudi Arabia and Sudan.

**Results:**

The outbreak in Saudi Arabia caused 883 human cases, with a case fatality rate of 14% and more than 40,000 dead sheep and goats. In Sudan, 698 human cases of RVF were recognized (case fatality, 31.5%), but no records of affected animals were available. The ecology and environment of the affected areas were similar with irrigation canals and excessive rains providing an attractive habitat for mosquito vectors to multiply. The outbreaks resulted in livestock trade bans leading to a vast economic impact on the animal market in the two countries. The surveillance system in Sudan showed a lack of data management and communication between the regional and federal health authorities, while in Saudi Arabia which is the stronger economy, better capacity and contingency plans resulted in efficient countermeasures. Studies of the epidemiology and vectors were also performed in Saudi Arabia, while in Sudan these issues were only partly studied.

**Conclusion:**

We conclude that a One Health approach is the best option to mitigate outbreaks of RVF. Collaboration between veterinary, health, and environmental authorities both on national and regional levels is needed.

The emerging Rift Valley fever (RVF) is a transboundary zoonotic disease, transmitted to animals and humans via mosquito bites, but also directly through exposure to blood, body fluids, or tissues of infected animals (1). It is caused by RVF virus (RVFV) belonging to the *Bunyaviridae* family, genus *Phlebovirus*, which is endemic in many African countries, and also since 2000 in the Arabian Peninsula where Saudi Arabia and Yemen have been affected (2, 3). The RVFV usually causes outbreaks after floods when the conditions are favorable for virus transmission via mosquitoes (4). There is great concern that the disease will continue to spread to new regions around the world, such as South-East Asia, Americas, and Europe with potentially devastating consequences (5–7). The increased animal trade and the possibility of vectors transported aerially as well as climate change could increase the risk for the disease to expand further (8–10).

RVFV infection of livestock causes abortions and high perinatal mortality (>95%) in herds of sheep, goats, cattle, and camels. These animals are important not only for meat production but also for dairy production and trade. Outbreaks devastate the local economy, result in export embargoes, and contribute to poverty in many regions of the affected countries. Human infection may be severe and historically the disease has had a case fatality rate in humans of 1% (11), but in recent outbreaks rates of 14–31% have been reported (12–14). Hepatorenal failure and hemorrhagic, ocular, and encephalitic complications were found in severe cases (12, 15, 16). The putative increase of severity and the recent geographical expansion have put the focus on the emerging nature of RVF. Notably, the epidemiological aspects of RVF, which include ecology, environment, knowledge, practices, and both animal and human health, emphasize the necessity of a One Health, One World approach (17). This concept/approach implies that multidisciplinary countermeasures in a global context have to be introduced to ensure better environment, better food safety and protection of human livelihood. In this context, it is important to study how a RVF outbreak is affecting different regions and countries.

RVF is a transboundary disease, which has an impact on the important livestock trade between Sudan and Saudi Arabia, separated only by the Red Sea, where Sudan is the exporting and Saudi Arabia is the importing country. The close economic links, the geographical proximity of the two countries, and the two recent important outbreaks of RVF in Saudi Arabia 2000 and Sudan (2007) motivated us to investigate these outbreaks from a One Health perspective. Specifically, we investigated the reasons for, the consequences of, and countermeasures against the outbreaks. The study revealed similarities and discrepancies between the two countries regarding outbreak causes, consequences, epidemiology of the disease, and how the RVF outbreaks were tackled in terms of a One Health approach at the human–animal interface.

## Methods

Using the terms ‘RVF’, ‘RVFV’, ‘Saudi Arabia’ and ‘Sudan’, 95 articles were identified by searching Medline through PubMed (www.ncbi.nlm.nih.gov/sites/entrez) and after reading their abstracts, 37 articles were found relevant and included in this review. The books, reports, and fact sheets that include information about the disease were searched through the following sources: World Health Organization (WHO) (www.who.int); World Organization of Animal Health (OIE) (www.oie.int); Food and Agriculture Organization of the United Nations (FAO) (www.fao.org); Saudi Arabia Ministry of Health, Google scholar; and United States Center for Emerging Issues (www.aphis.usda.gov).

## Results

### Outbreak and consequences

#### Saudi Arabia

Saudi Arabia experienced a large RVF outbreak in the year 2000. It was the first outbreak in this country and the first outside Africa. The first reports on animal disease, reminiscent of RVF, were in August and early September with widespread abortions in sheep and goats (18). The appearance of RVF in ruminants was in some cases dramatic with 60–90% abortions in pregnant animals within a period of 10–14 days (18). In dryer zones in the north, only 5–20% of the pregnant animals were affected (18). During these first days of the outbreak, 2,699 abortions and 943 deaths were recorded in the animals (18). It was later estimated that during the outbreak around 40,000 animals died such as sheep, goats, camels, and cattle and 8,000–10,000 of them were aborted (19). Human cases with RVF was reported in Saudi Arabia for the first time in September 10, 2000 (20), when the Ministry of Health in Saudi Arabia asked for help to investigate unexplained human hemorrhagic fever cases reported from the health care centers of the western border in association with animal deaths. The serum samples of the suspected human and animal cases were confirmed to be RVF by the United States Centre for Disease Control and Prevention (CDC) 5 days later. In total, the outbreak lasted for about 7 months and 883 human cases were recorded, with 124 deaths (case fatality rate, 14%) in Saudi Arabia (3) and an additional 1,328 human cases, with 166 deaths, were concurrently recorded in neighboring northwestern Yemen (21–23). After the outbreak was declared, a team was established in collaboration between the Ministries of Health, Agriculture and Water, and the Ministry of Municipalities and international organizations such as CDC, WHO, and National Institute of Virology, South Africa, to control the outbreak (2, 20, 22).

**Fig. 1:**
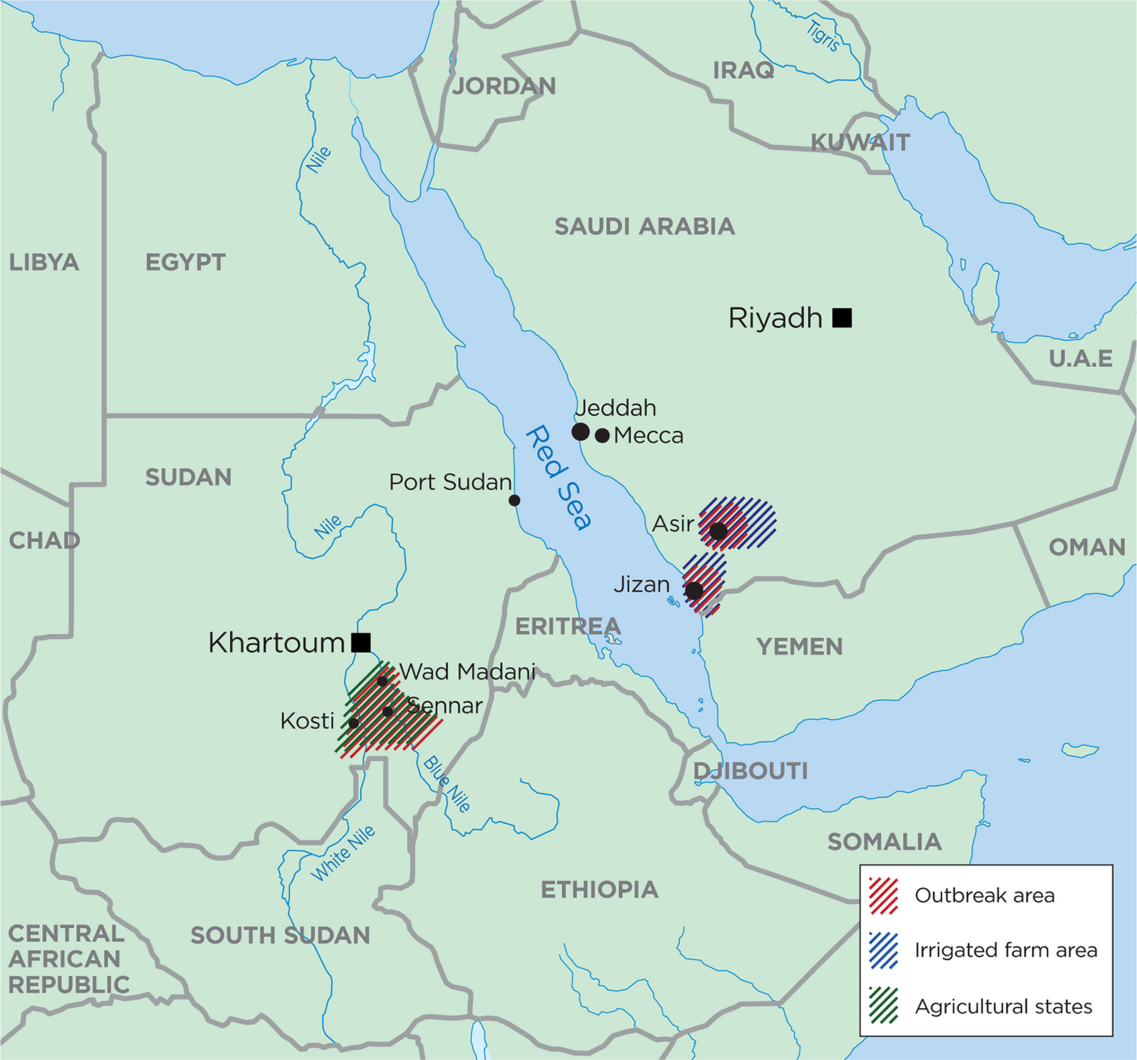
Map of Sudan and Saudia Arabia. RVF outbreak areas are indicated in red, irrigation and/or areas with seasonal water are in blue, and agricultural states in green.

The area first affected by the disease was Jizan province with pockets of the disease in Asir and Alqunfdah (Fig. 1). The majority of animal cases were in the Jizan region (66%), with 27% in Tahamat Asir and 7.5% in Tahamat Makkah (23). The seroprevalence in humans was 47% in Jizan, 48% in Asir, and 4% in Alqunfdah (20, 21) (Fig. 1). Jizan province is located in the south west of the country near the coast of the Red Sea bordering Yemen where an outbreak occurred simultaneously, and also for the first time (20, 21). The RVF outbreak in Saudi Arabia started in the western part, with the largest impact in the Jizan region. This could be the result of an amplification of an already previously introduced virus when the conditions were favorable for transmission (24). The outbreak region of Jizan and the surrounding area was located in the valley called Tihama Wadi, which covers most of the coastal plain of Saudi Arabia in the southwestern area. The rainfall in the surrounding mountains is transported by seasonal water to these plains (18) and has encouraged the people to reclaim the land for cultivation with the use of traditional irrigation canals (18). In 2000, there was a rainier season compared to the normal annual rainfall in this area (25).

#### Sudan

The Sudanese outbreak in 2007 followed unusually heavy rains resulting in severe floods (26). The first human RVF cases appeared at the beginning of September 2007 (16, 27). Then, in October 18 2007, the Federal Ministry of Health (FMoH) Sudan asked the WHO to assist in the investigation and control of a suspected hemorrhagic fever outbreak and after analysis of human patient samples, an RVF outbreak was declared 10 days later (14). Two weeks later, the Sudanese Ministry of Animal Resources and Fisheries informed OIE that positive cases of RVF had been found in cattle and sheep at the end of October in White Nile state at the same place where human cases were reported (28, 29). Later, RVFV positive goats were detected (30). A task force from FMoH and Federal Ministry of Animal resources and fisheries started to deal with the outbreak with assistance from the Eastern Mediterranean Regional Office of WHO in terms of technical support (31). The outbreak lasted until January 2008. During the outbreak, 698 human cases of RVF were recognized with 222 deaths (case fatality rate: 31.5%) (32). No records of the total number of cases in animals are available.

The most severely affected areas were three agricultural states in central Sudan near the White Nile and the Blue Nile (White Nile, Sennar, Gazira) (Fig. 1) (31). Interestingly, the virus was detected here already in 1973 during a previous RVF outbreak close to Kosti in the White Nile state (Fig. 1) (33). It has been suggested that infected animals passing Gazira state introduced and caused the outbreak close to Wad Madani on their way to Khartoum or to Port Sudan in the northeast (24, 34). According to WHO, the majority of the human cases were seen in Gazira (28), an area with agriculture and irrigation canals. Many RVF cases were also detected in Khartoum, but it was suggested that patients were infected in rural areas (35).

### Strategies for control

#### Operationalization of One Health in Saudi Arabia

After the outbreak was declared in Saudi Arabia, control measurements were simultaneously implemented toward both humans and animals. Surveillance was urgently introduced to detect cases of RVF among ungulates and humans. Dead animals were disposed of in an appropriate manner (2, 22). A survey among animals was also conducted to locate target areas for animal vaccination and then apply a vaccination campaign that started in October 2000 (19, 20, 23). Around 1,200,000 doses of vaccine were reported to be imported to Saudi Arabia and the campaign continued during 2001 with more than 10 million ruminants being vaccinated (23). This was accompanied by a restriction on animal movements outside the affected areas and a ban on animal imports from RVF-enzootic countries (21). For human health, a detailed case definition was described and distributed to all health providers by the Saudi Ministry of Health (21). Two laboratories (one in the affected regions and the other in the capital of the country) were well prepared for diagnosis of RVFV antibodies in suspected cases (2). Epidemiological investigation was performed to identify risk factors (22). Furthermore, training sessions were held to inform medical health providers on how to manage the suspected cases clinically (20). In addition, an entomological study to search for the mosquito breeding grounds (22) was followed by an intensive mosquito control program with spraying (20, 36). The One Health strategy implemented by Saudi Arabia on both the animal and human side succeeded to limit the effect of the outbreak and curb the disease to the onset area of the Gizan region. Only sporadic cases have been recorded in Saudi Arabia since the outbreak and only in the same regions as the original outbreak (19). It is difficult to rate the effect of the control measures, and it is rather the integrated control measures that were successful.

#### Operationalization of One Health in Sudan

As soon as outbreak was announced, the governors of the affected states formed a committee to deal with the outbreak. The committee aimed to reflect a One Health strategy to tackle the disease outbreak. The committee was led by the general director of the government in the affected states and consisted of members from the ministry of Health, ministry of animal resources and a consortium of consultants, including community medicine, internal medicine, ophthalmology, nephrology, and pharmacology.

For control of the RVF outbreak in Sudan, the committee aimed to target both human and animal health. To minimize the risk of transmission in slaughterhouses, strong precautions were applied (37). Unfortunately, no information was available about how dead animals were disposed of. Restrictions of animal movement were introduced. Normally, the system of pasture in Sudan includes free movement of herds over large distances. To assess the situation, surveillance of animal cases in the most affected states (Gazira, Sennar, and White Nile) was performed (38, 39). Vaccination of animals at high risk was implemented at the later stages of the outbreak in other states, such as Upper Nile state, south of White Nile state (37–39). To control the vectors, insecticides were used among animals and in the environment (38, 39). Active surveillance was initiated to find human cases as quickly as possible. Moreover, public health actions such as social mobilization of the society were implemented (26–28). NGOs were involved in health promotion and volunteers from the local communities were trained to be a part of the health promotion against the disease. There was an attempt to enhance the diagnostic capacity in terms of trained technicians and availability of laboratory supplies to detect the cases (28).

Although Sudan aimed to implement a One Health strategy as mentioned above, it did not restrict the disease to the onset region in White Nile state. It spread to the states of Gazira, Sennar, and Kassala. The difference regarding the implementation of One Health in Sudan and Saudi Arabia is intriguing and possibly reflects the difference in resources.

## Discussion

### Virus transmission

The RVF outbreaks were unexpected and caused great commotion in both Saudi Arabia and Sudan. No RVF outbreaks had been recognized for more than 30 years in Sudan and RVF had never been present in Saudi Arabia. The transmission to Saudi Arabia is suggested to be from Eastern Africa by importation of infected animals (40), similar to the suggested route of introduction of RVFV to Egypt in 1977 from Sudan (41). The virus causing the Saudi Arabia outbreak belonged to the same strain that caused the 1997–98 outbreak in the African Horn (42) and the virus could either have been a previously unrecognized introduction (43) followed by virus endemicity in the Wadi zones for some years in cryptic foci (19) or a very recent transmission. As mentioned above, Sudan has a history of RVF outbreaks although no major outbreaks have been reported for a long period. Similar to Saudi Arabia, the outbreak in Sudan could have been caused by an enzootic virus present for a long time (33, 44) or it could have been an introduction of RVFV from the large outbreak in Kenya, Somalia, and Tanzania in 2006–07 that preceded the Sudan outbreak. Recently, sequencing of RVFV strains from the 2007 outbreak indicated that genetic RVFV variants circulating in Sudan were all related to sublineages from the 2006–07 eastern Africa epizootic (45). However, the sequencing suggested an earlier common ancestor from 1996 (45) coinciding with the 1997–98 outbreak in the African Horn (42), so multiple introductions are likely (45).

### Climatic and ecological aspects

In Sudan, the outbreak was triggered by severe flooding (26) as a result of El Niño conditions and increasing surface temperature of the eastern Pacific Ocean (24). This climatic condition also affected Kenya, Tanzania, and Somalia during the 2006–07 outbreaks (24). This extreme weather was important but most probably not the only reason, because such weather has occurred before in Sudan without any documented outbreaks. In Saudi Arabia, the outbreak coincided with heavy rains in the affected and surrounding areas (18). The area of most interest in terms of rainfall patterns could be the mountain catchment areas that feed the Wadi that constitute the alluvial floodplains in the region (18). This mountain area had extensive rains during the 2000 rainy season, filling swamps and wetlands during 2000, which could provide good conditions for mosquito breeding (18). Such an area is the Tihama Wadi in Saudi Arabia where many irrigation canals are present (15). For Sudan, remote sensing of environmental factors predicted that conditions likely to lead to an RVF outbreak were found 3 months before the outbreak (24). The prediction was generalized and did not take into account regional differences, which resulted in an overall performance of 50% of the human cases mapped to the correct locations (24). This indicates the need for higher resolution models (24, 34). The ecology of the area could be more or less suitable for an outbreak to occur. In Sudan, agricultural areas around the tributaries of the Nile, such as Gazira, were affected by the outbreak while the Saudi affected areas were near the coast of the Red Sea, connected to irrigation farming (Fig. 1). The ecology and environment in the affected regions of the two countries were similar providing an attractive habitat for the mosquito vectors to multiply, similar to the Ifakarra rice valley in Tanzania and the highlands of Madagascar (24). Large-scale modifications of the environment could also lead to better conditions for RVFV vectors. The first outbreak of RVF in Egypt in 1977 was triggered by the construction of the Aswan dam on the Nile River, starting mosquito breeding in newly flooded areas. In 1987, the Mauritanian RVF outbreak was caused by flooding after the Diama dam construction in the Senegal River, which later led to the spread of RVF in Mauritania (46, 47). It is worth mentioning that a new large dam was recently constructed in Merowe on the Nile river basin in north Sudan. Around this area there was serological evidence of RVFV in the 80s (48). A future scenario could be similar to the scenario in Egypt 1977 where an outbreak occurred 6 years after the Aswan dam construction (46). Thus, surveillance of animals and mosquitoes around the dam is needed in order to detect the presence of RVFV as early as possible in order to disrupt any future outbreak in this area of the country.

### Vector competence and animal reservoir

During the outbreaks most efforts were directed toward acute control measures, but later, studies regarding vector competence of the RVFV were performed in Saudi Arabia (25). The result of these studies was important for later control and surveillance of mosquitoes for new RVF outbreaks (40). In Sudan, the role of vectors was only partially studied and only at the later stages of the outbreak (27). This weakens control efforts for future outbreaks and leaves the local epidemiology of the disease unclear. In the two countries the disease was restricted to rural areas. However, in Sudan the virus was also identified from some species of mosquitoes in Khartoum (27). It is unknown if the virus could be established and circulate in densely populated urban areas where surveillance should be considered in the future. In general, studies need to be implemented to identify RVF competent vectors at the country level. The better the understanding of the vectors and their preferred animal hosts, the more efficient and cost-effective the prevention and control programs. Cattle, sheep, and goats were affected in the outbreak in the two countries but other animals, such as rats, camels, and donkeys (49), could also be involved in the virus transmission cycle. The most severe form of the disease is usually found in animals of European origin, rather than local ones (14). More information about the breeds involved in the outbreaks will lead to a better understanding of the disease epidemiology and consequently facilitate proper prevention and control of the disease.

### The role of surveillance systems in detecting the RVF outbreaks

Saudi Arabia and Sudan are large countries with several neighboring countries. Shared borders might increase the risk of spreading RVF unless great care is taken regarding trade regulation of animals in the region. Application to regulations and guidelines from the World Trade Organization and OIE would reduce the danger of RVF being introduced to new unaffected countries. For both Saudi Arabia and Sudan, the surveillance system did not work properly to detect the virus in animals before it spread to humans, although it has been suggested that RVF is well established in animals before any disease outbreak in humans (50). In Saudi Arabia, disease occurrence in humans was reported in association with animal deaths while in Sudan the animal cases were discovered later. The period between the onset of an unknown febrile illness among humans and the confirmation of a RVF outbreak was 18 days in Saudi Arabia, while it took three times longer in Sudan (14, 20, 21). This discrepancy probably reflects the lack of data management and communication between the regional and federal health authorities in Sudan.

The outbreak in Saudi Arabia 2000 lasted for a longer time than in Sudan but with almost half the case fatality rate compared to Sudan. The stronger economy and the better capacity, contingency plans, and health care system in Saudi Arabia were probably the main factors for the lower fatality rate. One could suspect that there were more undiagnosed cases and late recognition of RVF cases in Sudan compared to Saudi Arabia, which could explain the higher case fatality rates associated with the human cases in Sudan. A similar experience of late diagnosis was seen in other RVF outbreaks, for example in Kenya in 1997–98 where 467 unexplained deaths occurred in humans before it was discovered that it was caused by RVF (51). In general, the recognition of human and animal cases needs good communication and collaboration between veterinary and health authorities to gather the epidemiological features of a zoonotic outbreak. In particular, surveillance and prediction models of imminent outbreaks could be improved by collecting data such as animal and human sera as well mosquitoes from high-risk RVF sites during interepidemic periods to detect virus activity as early as possible (52). At later stages of the outbreak in Saudi Arabia, results from studies undertaken earlier regarding risk factors were used in the health education messages and led to a reduction in morbidity. However, in Sudan the risk factors were not studied in detail, which probably led to a less cost-effective control strategy.

To advocate for surveillance in RVF endemic regions among affected countries, we have to think seriously to compensate the communities that are exposed to RVF outbreaks to enable them to recover from their economic losses. After verification of RVF cases by veterinary authorities and community leaders, compensation could, for instance, be in the form of replacing dead or sick animals with new animals, and vaccination against all vaccine preventable diseases including RVF. Part of the support should also be directed toward improving the infrastructure of the veterinary facilities in the affected areas. This support could be offered by the government of the affected country if its economy allows that. In addition, the neighboring countries could also contribute because they are also in danger when the disease expands. Furthermore, regional and international organizations should contribute to such a compensation program. It would also be beneficial if regional funds were set aside to use for combating emerging infectious diseases including RVF. Altogether, such actions would encourage local communities and governments to notify authorities earlier about the circulation of RVF in their countries.

### One Health economics of RVF

In Sudan, livestock is one of the most important contributors to the national economy. It employs around 40% of the population and contributes to 25% of the gross domestic product (GDP) (53). Before the RVF outbreak, Sudan's share of the total sheep trade in the world was 10% and the main export market was Saudi Arabia (54). Both the domestic and international animal market of the country has a huge economic impact. This was clearly demonstrated during the ban of animal trade from November 2007 to August 2008. Since most of the export animals came from the rural areas of Sudan, one could expect the ban to have the most severe impact on rural households and the rural economy. Unfortunately, no studies have been undertaken to explore that. The disease killed and caused abortions in many animals such as sheep and goats. They are an important source of food, milk, and meat, and this animal trading brings money into the rural communities in the affected regions. In Saudi Arabia, the estimated direct and indirect losses due to the RVF outbreak reached US $75–90 million in 1 year (19, 36). After the outbreak, the ban on importing animals from Eastern Africa disrupted the trade of live animals in the country. This trade is relevant to the religious festival events of Muslims in Mecca, Saudi Arabia, and it is estimated to be worth US $ 600–900 million annually. The impact was on both the demand side of Saudi Arabia and the main supplying countries in East Africa, including Sudan (54). Thus, the RVF outbreaks point to the severe consequences involved in disrupting the animal trade economy and the current lack of information regarding the economic impact of RVF at the macro- and microeconomic level. Because the outbreaks are first recognized at the local level, it is important to ensure cooperation with local communities for prevention and control efforts at an early stage. Therefore, studies regarding the social consequences of RVF outbreaks for rural communities and qualitative studies to investigate the knowledge and attitudes toward the disease are needed.

Because animals, humans, and the environment are all involved in the infection cycle of RVFV, we expect that the budgets of ministries representing these interests are compiled together to prioritize control strategies against RVF. Such an initiative has not been visible in Saudi Arabia or Sudan.

## Conclusions

Human and animal health is a result of the sustainable relationship between humans, animals, and the environment. Therefore, it is important to tackle health problems in animals to protect human health. To prevent future RVF outbreaks, information based on virologic, epidemiologic, and demographic data; surveillance; and a better understanding of environmental factors and the ecology of RVFV are required to fine-tune existing approaches that could predict an outbreak. The impact of climate change on the spread and expansion of the disease needs to be studied in detail in order to adapt mitigation strategies. To achieve the goal of One Health, good collaboration between international organizations on a planning level as well as veterinary and health authorities on the executive level in each country is needed; this could reduce the occurrence of RVF in Sudan, Saudi Arabia, and other countries where the disease could emerge.
